# Supplementary material for: Complexome profiling of the Chlamydomonas psb28 mutant reveals TEF5 as an early PSII assembly factor
Source: Plant Cell. 2025 Mar 17;37(6):koaf055. doi: 10.1093/plcell/koaf055 (PMC12164590; doi:10.1093/plcell/koaf055)
Supplement: koaf055_Supplementary_Data [file koaf055_supplementary_data.zip › psb28_interactive.zip!/plots/intensity/qualityPlots/peptides/_Cre11.g467400.t1.2.html]

### Cre11.g467400.t1.2

Trivial name:   
SeqCoverage [median]: 30.10  
Mass: 57.00 kDa  
  
 

| Sequence | MissedCleavage | Mass [Da] | wtIntensity | muIntensity | Score |
| --- | --- | --- | --- | --- | --- |
| AAAAAAAASAAPAAPAPAK | 0 | 1518.82 | 13905.14 | 6790.71 | 149.22 |
| AAATTAVVETK | 0 | 1060.58 | 13203.89 | 5111.87 | 156.92 |
| AAPAAAEPPAAAAPPAPPKEK | 1 | 1922.03 | 4296.90 | 872.58 | 106.57 |
| APAGAAAAAAAAK | 0 | 1010.55 | 4193.25 | 637.03 | 194.20 |
| ASGSAPAAAGASGASAAPSKPAAVK | 1 | 2052.06 | 1064.55 | 0.00 | 73.13 |
| ASSVSASASSLGR | 0 | 1178.59 | 4963.67 | 2472.84 | 163.90 |
| ASTDATVAR | 0 | 890.45 | 3411.36 | 486.95 | 106.04 |
| LSGNTPSALGAAAK | 0 | 1256.67 | 8349.69 | 2003.70 | 134.98 |
| LVVQDRPMNIGLSPK | 1 | 1665.92 | 1954.88 | 2983.35 | 77.75 |
| SAASAGPVSPPSK | 0 | 1154.59 | 408.35 | 0.00 | 110.38 |
|  |  |  | 55751.7 | 21359.0 |  |
